# Essential Trace Elements in Patients with Dyslipidemia: A Meta-analysis

**DOI:** 10.2174/0929867330666230428161653

**Published:** 2023-07-07

**Authors:** Cui-Ping Li, Yu-Xin Song, Zi-Jun Lin, Mei-Lin Ma, Lian-Ping He

**Affiliations:** 1 School of Medicine, Taizhou University, Jiaojiang, Zhejiang, 318000, China

**Keywords:** Selenium, zinc, iron, copper, chrome, manganese, cadmium, dyslipidemia

## Abstract

**Background:**

Lipid metabolism is a complex process that includes lipid uptake, transport, synthesis, and degradation. Trace elements are vital in maintaining normal lipid metabolism in the human body. This study explores the relationship between serum trace elements and lipid metabolism.

**Methods:**

In this study, we reviewed articles on the relationship between alterations in somatic levels of zinc, iron, calcium, copper, chrome, manganese, selenium, and lipid metabolism. In this systematic review and mate-analysis, databases such as PubMed, Web of Science, and China National Knowledge Infrastructure (CNKI), Wanfang was searched for articles on the relationship published between January 1, 1900, and July 12, 2022. The meta-analysis was performed using Review Manager5.3 (Cochrane Collaboration).

**Results:**

No significant association was found between serum zinc and dyslipidemia, while other serum trace elements (iron, selenium, copper, chromium, and manganese) were associated with hyperlipidemia.

**Conclusion:**

The present study suggested that the human body's zinc, copper, and calcium content may be related to lipid metabolism. However, findings on lipid metabolism and Iron, Manganese have not been conclusive. In addition, the relationship between lipid metabolism disorders and selenium levels still needs to be further studied. Further research is needed on treating lipid metabolism diseases by changing trace elements.

## 1 INTRODUCTION

Lipid metabolism is an essential and complex biochemical reaction in the body [1]. The biochemical reaction is the oxidation-reduction reaction of fat digestion [2], decomposition, absorption, and synthesis in the body with the help of various related enzymes [3]. It is processed into the substances needed by the body. Fat metabolism ensures the regular operation of human physiology [4] and is of great significance to life activities. In addition, lipids are essential substances for the body's energy storage [5] and energy supply and are also important structural components of biological membranes [6, 7].

Diseases caused by lipid metabolism disorders have become common and epidemic in modern society [8]. Lipid metabolism disorders include reduced high-density lipoprotein [9], elevated low-density lipoprotein, elevated total cholesterol, and elevated triglyceride levels. In addition, lipid metabolism disorders can cause diabetes, obesity [10, 11], cerebrovascular disease [12-14] and hypertension.

Although the content of trace elements in the human body is minimal [15], it has a powerful biological effect [16, 17]. They are involved in the metabolic processes of enzymes, hormones, vitamins [18], and nucleic acids [19], and their physiological functions mainly assist in the delivery of macro elements.

Recent studies have found that zinc, iron, selenium, copper, chrome, manganese, and calcium are closely related to lipid metabolism [20, 21]. Decreases in zinc, copper, and calcium affect normal lipid metabolism and lead to elevated serum total cholesterol and low-density lipoprotein levels. However, lipid metabolism and iron findings have yet to be conclusive and contradictory. Furthermore, trials on lipid metabolism disorders and selenium levels still need to be more definitive.

Disorders in lipid metabolism can cause life-threatening diseases that are now reaching epidemic proportions [22-24], which are associated with altered levels of trace elements. Therefore, a complete understanding of the disturbances in the ranks of micronutrients in the body is associated with lipid abnormalities appraisal.

This article's purpose is to review the existing and recent literature on the relationship between trace elements such as selenium, zinc, iron, calcium, chrome, manganese, selenium, copper, and dyslipidemia.

## 2 METHODS

This study was performed based on PRISMA (Preferred Reporting Items for Systematic Reviews and Meta-Analyses) and MOOSE (Meta-analyses Of Observational Studies in Epidemiology) guidelines [25].

### 2.1 Literature Search

In this study, databases such as PubMed, Web of Science and National Knowledge Infrastructure, and Wanfang databases were searched comprehensively for articles published between January 1, 1900, and July 12, 2022. We use the search terms “Selenium,” “zinc,” “iron,” “Copper,” “Chrome,” “Manganese,” “Cadmium,” AND “dyslipidemia [Majr]” OR “Hypercholesterolemia” OR “Hyperlipoproteinemias” OR “Hypertriglyceridemia.” The author reviewed relevant articles for inclusion. Exclusion criteria were studies that (1) did not include trace elements related to cases; (2) lack of data to be included in the Meta-analysis; and (3) were case reports, commentaries, dissertations, or reviews.

### 2.2 Inclusion/Exclusion Criteria

Papers that meet the following criteria include Original research papers written in English on trace elements and dyslipidemia. These include observational research articles describing human research. Non-original research articles, duplicate articles, review articles, case reports, and animal research are not included. In addition, all prospective cohort and case-control studies evaluating the effects of dietary zinc, iron, copper, chrome, manganese, selenium, and calcium intake on lipid metabolism were included. The provision of hazard ratios, relative risks, and odds ratios was considered a prerequisite for the inclusion of studies.

### 2.3 Risk of Bias Assessment

The risk of bias in the included studies was assessed by two authors using the ROBINS-I tool [26]. This tool consists of seven questions aimed at determining bias based on confounding, participant selection, exposure classification, bias due to departures from intended exposures, missing data, outcomes measurement, and selection of the reported result.

### 2.4 Statistical Analysis

The meta-analysis was performed using Review Manager 5.3 (Cochrane Collaboration). The standardized weighted mean difference (SMD) with a corresponding 95% confidence interval (CI) was used to compare the mean serum trace elements between the two groups. Heterogeneity among effect sizes was tested using a Q statistic and an I^2^ index. The Q statistic tests the null hypothesis that effect sizes are homogeneous in the analysis (Q=0) [27]. The funnel plot was applied to detect possible publication bias [28].

## 3 RESULTS

### 3.1 Literature Search

**Fig. (1):**
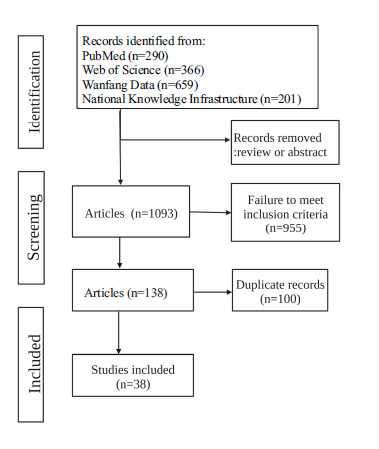
Flowchart of study selection.

After reviewing the abstracts, 332 cases were excluded as non-article for the format of review. Seven hundred nine abstracts were subsequently selected for scanning according to inclusion/exclusion criteria. Finally, thirty-eight articles were included in this paper to explore the relationship between serum trace elements (zinc, iron, copper, selenium, calcium) and lipid metabolism (Fig. **1**) [29-65].

### 3.2 Characteristics of Included Studies

Among those articles were thirteen studies on zinc, eight on iron, four on selenium, ten on copper, three on chrome, two on manganese, four on cadmium, and three on calcium (Table **1**).

### 3.3 Findings from the Meta-analysis on Zinc

**Fig. (2):**
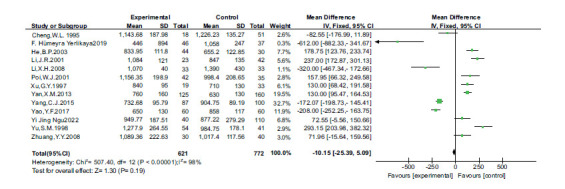
Forest plot of zinc and dyslipidemia.

**Fig. (3):**
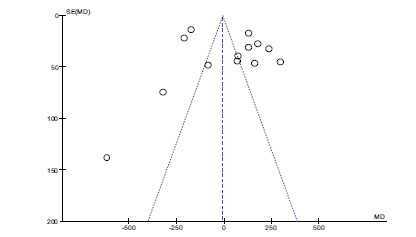
Funnel plot of zinc and dyslipidemia.

There are no statistical differences between the two groups (*P* > 0.05, Fig. **2**). Funnel plots determined publication bias. There was no evidence of publication bias (Fig. **3**).

### 3.4 Findings from the Meta-analysis on Iron

**Fig. (4):**
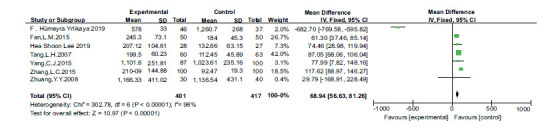
Forest plot of iron and dyslipidemia.

**Fig. (5):**
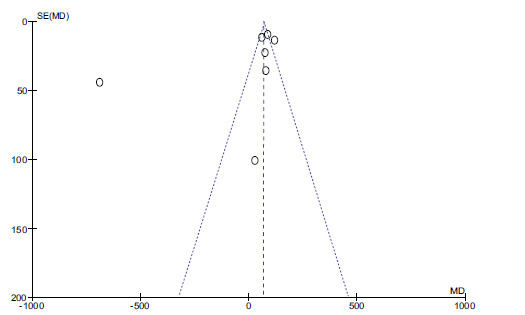
Funnel plot of iron and dyslipidemia.

The difference between the two was statistically significant. (*P <* 0.05, Fig. **4**). Publication bias was also assessed by funnel plots. There was no evidence of publication bias (Fig. **5**).

### 3.5 Findings from the Meta-analysis on Selenium

**Fig. (6):**
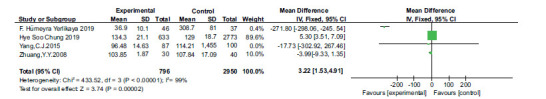
Forest plot of selenium and dyslipidemia.

**Fig. (7):**
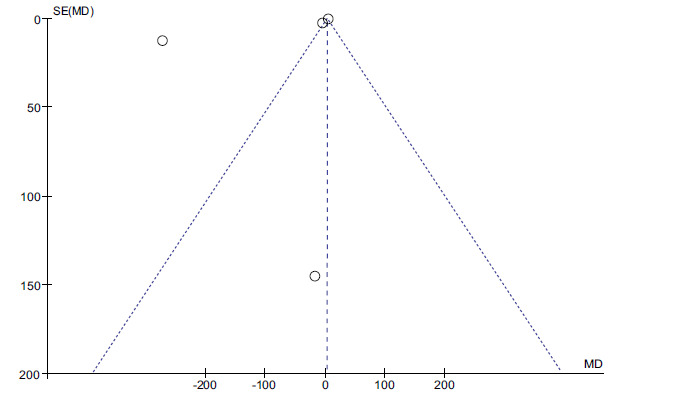
Funnel plot of selenium and dyslipidemia.

The difference between the two groups is statistically significant (*P* < 0.05, Fig. **6**). We also evaluated potential publication bias by constructing Funnel plots. There was no evidence of publication bias (Fig. **7**).

### 3.6 Findings from the Meta-analysis on Copper

**Fig. (8):**
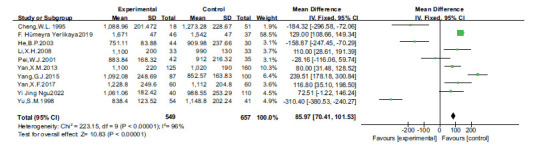
Forest plot of copper and dyslipidemia.

**Fig. (9):**
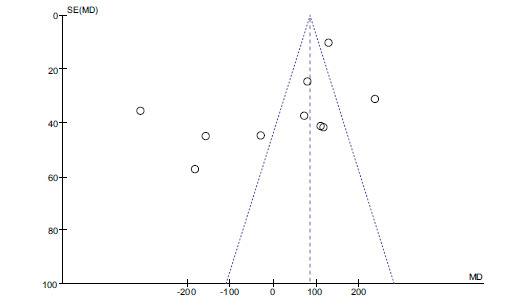
Funnel plot of copper and dyslipidemia.

There is a statistically significant difference (*P <* 0.05, Fig. **8**) between the two genotypes. Publication bias was evaluated by using funnel plots. There was no evidence of publication bias (Fig. **9**). 

### 3.7 Findings from the Meta-analysis on Chrome

**Fig. (10):**
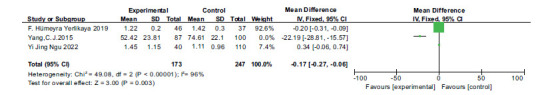
Forest plot of chromium and dyslipidemia.

**Fig. (11):**
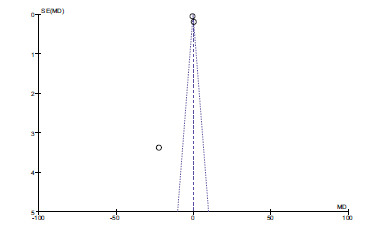
Funnel plot of chromium and dyslipidemia.

There is a statistically significant difference between the two groups (*P <* 0.05, Fig. **10**). Funnel plots determined publication bias. There was no evidence of publication bias (Fig. **11**).

### 3.8 Findings from the Meta-analysis on Manganese

**Fig. (12):**
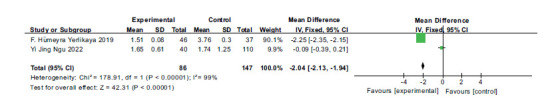
Forest plot of manganese and dyslipidemia.

**Fig. (13):**
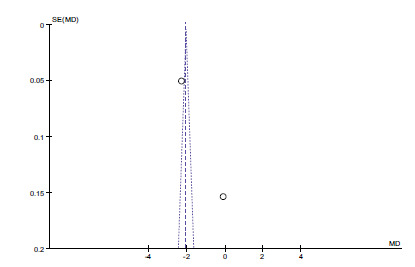
Funnel plot of manganese and dyslipidemia.

Since *P <* 0.05 (Fig. **12**), the difference between these two groups is statistically significant. Publication bias was assessed using funnel plots. There was no evidence of publication bias (Fig. **13**).

## 4 DISCUSSION

### 4.1 Zinc

Zinc contributes to the biochemical and metabolic processes of carbohydrates [66], proteins, and lipids in humans. In addition, zinc is a critical component of multiple enzymes and transcription factors involved in the pathophysiology of obesity [31, 67, 68]. Serum Zinc-alpha2-glycoprotein (ZAG) is a lipid-mobilizing adipokine that impacts lipid metabolism [69], glucose metabolism, and the regulation of insulin sensitivity. Zinc is an essential regulator of ZAG homeostasis [70]. Therefore, altered zinc metabolism in obese individuals may impair ZAG function.

Numerous studies have shown that zinc [71] and zinc-related adipokines play an important role in lipid metabolism. Zinc and ZAG were negatively correlated with body weight, BMI, and adipose tissue. A study by Rios-Lugo [67] and colleagues from Mexico found a decrease in serum zinc levels in overweight and obese individuals (*P* < 0.05) and a negative correlation between BMI and serum Zinc levels (r =-663 and *P* < 0.001). Multiple studies have found that zinc supplementation significantly reduced plasma LDL-C, TC, and TG [72], potentially reducing the risk of cardiovascular morbidity and mortality among patients with obesity. In addition, ZAG treatment was found to increase levels of fat triglyceride lipase and hormone-sensitive lipase [73], which contribute to the inhibition of lipogenesis and enhancement of lipolysis. Zinc transporters or factors associated with zinc homeostasis may be candidate biomarkers or therapeutic targets [74]. However, the mechanism of their impact on lipid metabolism is still controversial, and further research is needed.

To summarize, decreased serum zinc levels and impaired ZAG function lead to disturbances in fat metabolism. Further, analyses are required to regulate fat metabolism by zinc transporters and therapies against obesity.

In this meta-analysis, our research indicates no significant association between serum zinc and hyperlipidemia. The results are different from the previous results of a meta-analysis. This discrepancy could be attributed to some reasons. Our research needs to distinguish the type of blood fat, which includes total cholesterol (TCHO), triglycerides (TG), high-density lipoprotein cholesterol (HDL), and low-density lipoprotein cholesterol (LDL). For example, it is reported in some existing literature that zinc supplementation lowered HDL levels, potentially increasing the risk of cardiovascular disease [75]. To continue exploring the relationships between serum zinc and dyslipidemia, we anticipate several improvements that will further refine the classification of blood lipids.

Besides, most of the study data come from China, which means our data may be skewed by region, ethnic lines, dietary habits, and so on. This concept will be explored by further increasing the sample size.

### 4.2 Iron

Iron, one of the essential trace elements in the body [76], participates in lipid metabolism in various forms. The two metabolic pathways, iron and lipid interact in many places in the body [77].

Iron can directly affect the distribution, fate, and secretion of lipids through the composition of enzymes and transporters involved in lipid metabolism [78]. Lipid metabolism can also be indirectly affected by the induction of oxidative stress and inflammation by iron in the ferrous form. Additionally, lipid metabolism also affects iron absorption and distribution to varying degrees. Zhao *et al.* [79] found a significant correlation between iron deficiency and obesity in studies without the ferritin-based diagnosis. This study revealed that obesity is significantly associated with iron deficiency. However, a survey by Wang *et al.* [80] found that obesity may be associated with systemic iron overload. This study sheds new light on the pathogenesis of iron and obesity. A cross- sectional study from the China Health and Nutrition Survey revealed that serum ferritin (SF), transferrin (TRF), soluble transferrin receptor (sTfR), and hemoglobin (Hb) [81], as markers of iron status, were significantly associated with the incidence of apolipoprotein abnormalities and SF concentrations were positively correlated with lipid ratio levels.

Additionally, iron is a driver of atherosclerosis and acts as a pro-oxidant [82, 83], causing lipid oxidation and tissue damage. Iron metabolism mediates atherosclerotic macrophage inflammation and lipid processing. Therefore, intracellular macrophage iron may be a promising drug target for the prevention of atherosclerosis [84, 85].

In conclusion, studies suggest that excess body weight may be caused by iron deficiency [45]. However, some research also indicates that obesity may be associated with excess iron [81]. The relationship between lipid metabolism and iron metabolism is unclear, and the underlying mechanisms require more in- depth studies. The regulation of iron metabolism and iron homeostasis may become a new treatment for lipid metabolism disorders and coronary arteriosclerosis.

In this meta-analysis, we discovered that the mean serum iron level in the dyslipidemia group was higher than in the control group. The results are consistent with previous results of the study [41]. In this meta-analysis, we don’t intake interference experiment. However, a recent study confirmed that high iron is positively correlated with the incidence of hypertension. Oxidative stress caused by high iron leads to elastin's destruction and promotes atherosclerosis [86]. Secondly, improving or eliminating iron overload, such as using iron chelators, can reduce the symptoms of hyperlipidemia and achieve the purpose of early prevention and treatment.

### 4.3 Selenium

Selenium is one of the indispensable trace elements in the human body [87, 88]. Selenium is in the active center of glutathione peroxidase 1 (GPX1) [89]. It is a cofactor of the blood glutathione peroxidase (GdSH-PX) [90], which can catalyze the production of hydrogen peroxide and organic hydroperoxide reduction. GPX1 is a selenium-dependent enzyme that reduces intracellular hydrogen peroxide and lipid peroxides [54, 91].

Cross-sectional studies in the United States, China, the Netherlands, and other countries have suggested that high circulating selenium concentrations are associated with a higher risk of dyslipidemia [55, 92, 93]. Elevated serum selenium levels were associated with elevated serum total cholesterol, LDL-C, HDL-C, apo B, and apo A-I [94]. However, the experimental results of the effect of selenium supplementation on lipid metabolism are inconclusive. In a longitudinal study of Chinese, Chen and his colleagues [56] found that individuals in the highest selenium quartile group showed a 1.11 stable disease (SD) decrease on TC (*P*<0.001), 0.41 SD increase on HDL-C (*P*<0.001) and 0.52 SD decrease on triglyceride after seven years than those in the lowest selenium quartile group. Furthermore, the study found that selenium had a modest beneficial effect on blood lipid levels in people with relatively low selenium levels.

In contrast, long-term zinc supplementation did not affect plasma cholesterol in the elderly in Denmark [57] and selenium supplementation in pregnant women may be associated with increased umbilical cord blood triglyceride levels [58]. In addition, overproduction of GPX1 may be beneficial in the event of diabetes or obesity. However, excessive GPX1 activity is detrimental to average glucose and lipid metabolism.

Overall, results in human studies regarding the relationship between selenium and lipid metabolism have needed to be more consistent, even conflicting. In addition, large randomized controlled trials must confirm whether excess selenium and GPX1 may contribute to dyslipidemia.

Our meta-analysis shows that the serum selenium level in the dyslipidemia group was higher than in the control group. The results are in line with previous studies [41]. As an essential component of glutathione peroxidase, selenium has the physiological function of removing lipid peroxides from the body [43]. Selenium deficiency can lead to the decrease of prostacyclin in the body, which is the accumulation of TC in the arterial wall, and promote the formation of AS, which in turn leads to the increase of TC, TG, LDL and the decrease of HDL [86]. After that, we will further improve the relationship between the two.

### 4.4 Copper

Copper is essential for some physiological functions of the human body [95], such as the formation of connective tissue and the support of the immune system.

Copper plays a crucial role in fat metabolism [96]. Copper deficiency (CuD) affects lipid metabolism, such as increased serum lipid levels. CuD is associated with disorders related to dyslipidemia, including obesity, cardiovascular disease (CVD), and nonalcoholic fatty liver disease (NAFLD) [97-99]. A series of studies have shown that sub-ideal levels of copper can exacerbate dyslipidemia and increase levels of oxidative stress. Numerous studies have suggested that marginal CuD is an underlying etiology of disease features such as NAFLD by disrupting lipid metabolism [100-102]. Although copper is essential for the breakdown of fat cells, high blood copper levels are associated with obesity, and serum copper levels are positively correlated with BMI, leptin, and insulin [96, 103].

Further study of the specific pathways linking copper metabolism and apolipoproteins will help better understand the relationship between copper and lipids. It is also necessary to continue to explore the pathogenesis of copper and copper transporters, such as copper- transporting p-type adenosine triphosphatase 1 (ATP7A) [104] and 2 (ATP7B) [103, 105], in lipid metabolism disorders. Furthermore, potential therapeutic strategies for copper-lipid-related neurodegeneration also require further development.

Our research shows that the serum copper level in the dyslipidemia group was higher than in the control group, which is consistent with the previous result [30]. Studies have shown that the content of Cu in patients with hyperlipidemia, hyperglycemia, hypertension, and obesity is higher than that in ordinary people [86]. When the human body lacks copper, the serum cholesterol level increases significantly, and the low- density lipoprotein concentration rises abnormally. The cholesterol can return to normal after copper supplementation [30]. Because the types of blood lipids were not distinguished, the data were, to a certain extent, different. After that, we will improve the relevant issues and the meta-analysis.

### 4.5 Chrome

Chromium is an essential mineral that has a beneficial role in regulating insulin action, metabolic syndrome, and cardiovascular disease [106]. Studies show that chromium is an essential factor in enhancing insulin activity. After absorption in the gastrointestinal tract, chromium is most likely transported to cells bound to the plasma protein transferrin. This oligopeptide, combined with four chromium (III) atoms, forms uromodulin, which is essential for amplifying the insulin signaling effect [107].

Many animal experiments and clinical trials have shown that trivalent chromium can affect sugar metabolism and is an essential trace element for humans and animals. The physiological function of chromium is mainly the role of trivalent chromium, which exists in glucose tolerance factor (GTF) and acts as an active ingredient. Trivalent chromium is primarily involved in the synthesis and metabolism of carbohydrates, proteins, fats, nucleic acids, and amino acids, helps maintain the average glucose content allowed in the body and promotes the synthesis of hemoglobin. Chromium can also inhibit the synthesis of fatty acids and cholesterol, thereby reducing the effects of triglycerides, cholesterol, and low-density lipoprotein, so it is necessary for glucose metabolism and lipid metabolism [108].

In this meta-analysis, we discovered that the serum chrome level has an association with dyslipidemia, which is no different from previous research [29]. However, most of our citations come from China, which may cause data differences due to regional race and other reasons. In addition, there are relatively few references in this part, and the data are quite different, which will also affect the results to a certain extent. So, latterly we will further upgrade the data, enhance the sample size and reduce the errors.

### 4.6 Manganese

Manganese (Mn) is an essential trace element, necessary for the development and growth of the organism. The adequate content of this element in the body determines proper metabolism of amino acids, cholesterol and carbohydrates [109]. Mitochondrial dysfunction is both a contributing mechanism and complication of diabetes, and oxidative stress contributes to that dysfunction. Mitochondrial manganese-superoxide dismutase (MnSOD) is a metalloenzyme that provides antioxidant protection. MnSOD plays a critical role in protecting the mitochondria from free radicals normally generated during respiration by converting superoxide anions into hydrogen peroxide, which is detoxified into water by mitochondrial glutathione peroxidase [110].

Mn-containing polypeptides such as arginase and Mn-containing superoxide dismutase play essential roles in enzyme activities and oxidative stress. Insufficient Mn intake may have harmful health effects. Animal studies have found a link between dietary Mn and metabolisms of amino acids, lipids, proteins, and carbohydrates, suggesting that dietary Mn intake may be associated with some components of the Mets [111].

Our meta-analysis showed that serum manganese was higher in the hyperlipidemia group than in the control group. The results are consistent with previous results of the literature [34]. However, there needs to be more literature on serum manganese and hyperlipidemia, and only some data can be included in the analysis. If there are more related studies in the future, we will timely include the examination and improve the data.

### 4.7 Calcium

Calcium is the most abundant cation in the human body, and it can maintain the activity of nerves and muscles and promote the activity of certain enzymes in the body, such as adenosine triphosphatase and lipase [112].

Elevating intestinal calcium reduces serum cholesterol and TG, possibly by chelating cholesterol and bile acids. Administration of vitamin D or increased sensitivity to vitamin D increases serum cholesterol levels. Increased calcium intake and vitamin D levels in humans were inversely associated with body weight and fat [63, 114]. However, intervention studies on the effects of dietary calcium and vitamin D status on body fat mass and body weight are inconclusive. The relationship between calcium and CVD has been explored for a long time [115]. Studies examining the effects of calcium intake or calcium supplementation on cardiovascular risk have shown that systolic blood pressure is elevated at low calcium intake and decreased with increased calcium supplementation. Lower calcium intake is associated with an increased risk of stroke [116]. However, the effect of calcium supplementation on stroke risk is unclear. Calcium supplementation may increase the risk of myocardial infarction.

## 5 STUDY LIMITATIONS

This study attempts to provide an overview of trace element intake and lipid metabolism. However, there are some limitations. The main constraints are the misclassification of exposures and limited access to the databases. Therefore, we cannot draw any specific conclusions. To reduce the impact of this flaw, we chose PubMed because it contains multidisciplinary peer- and non-peer-reviewed literature, respectively. The diseases caused by metabolic disorders are diverse, and it is not sufficient to state only obesity and overweight. When trace elements affect lipid metabolism through various mediators and enzymes, the article does not discuss enough in this section. Exposure to biomarkers can be a problem because various physiological and pathological aspects affect the absorption, metabolism, and bioavailability of trace elements in organisms. In addition, many trace elements affect lipid metabolism. Due to limited space, other lipid metabolisms are not discussed in this review.

## CONCLUSION

The review suggests that trace elements are involved in lipid metabolism. Reductions in zinc, copper, and selenium lead to increases in serum total cholesterol and low-density lipoprotein levels. On the other hand, being overweight is associated with higher copper levels. The relationship between lipid metabolism and iron levels requires further investigation, as some studies link lipid metabolism disorders and obesity to iron deficiency, while others link it to iron excess. Likewise, although studies have found that disorders related to calcium signaling affect lipid metabolism, the nature of the link between lipid metabolism and *in vivo* calcium levels remains to be elucidated. The causal relationship between altered levels of trace elements *in vivo* and lipid metabolism is unclear. More in-depth studies are still needed in this field to explore the complex mechanisms of the relationship between lipid metabolism and trace elements.

## AUTHORS’ CONTRIBUTIONS

Mei-Lin Ma, Zi-Jun Lin and Lian-Ping He contributed to the conception of the study, Mei-Lin Ma and Yu-Xin Song wrote the manuscript, Lian-Ping He and Yu-Xin Song contributed to the revision of the manuscript. All authors approved the final manuscript for submission.

## Figures and Tables

**Table 1:** Table shows the relationship between trace elements and lipid metabolism.

**Trace Element**	**References**	**Country**	**Study Design**	**Study Aim**	**No. of Participants**
Zinc	Ngu, Y. J. [29]	China	A cross-sectional study	To clarify associations of exposure to essential and non-essential metals with body composition and risks of obesity and MetS.	150 Taiwanese adults (100 men and 50 women)
	Li, X. H. [30]	China	A cross-sectional study	To further clarify the correlation between blood glucose and dyslipidemia and the levels of serum zinc, copper, and magnesium in type 2 diabetes mellitus patients.	33 diabetes mellitus patients and 33 non-diabetes mellitus healthy controls
	Laura C. [31]	Italy	A prospective cohort study	The study aimed to clarify the influence of dietary zinc intake on some metabolic, inflammatory, and zinc status parameters in adult overweight/obese subjects.	Total (n) = 223 (n=100) with low-zinc dietary intake (n=123) with normal-zinc dietary intake
	Dennis C. [32]	China	A prospective cohort study	This study aimed to address the relationship between serum Zinc-alpha2-glycoprotein (ZAG) levels and adiposity and cardiometabolic risk factors in humans.	Total (n) = 258 (n=138) females (n=120) males
	Trine M. [33]	Denmark	A prospective cohort study	This study aimed to investigate the zinc transporter ZIP14 (SLC39a14) expression in human adipose tissue before and after weight loss and the regulation of ZIP14 during early adipogenesis.	Total (n) = 28 (n=14) obese individuals (n=14) non-obese individuals
	Yerlikaya, F. H. [34]	America	A cross-sectional study	The study aimed to investigate the relationship between plasma microRNA expression levels, which are associated with lipid metabolism, and serum trace element levels in patients with primary hyperlipidemia.	Total (n) = 83 (n=46) obese individuals (n=37) non-obese individuals
	Cheng, W.L. [35]	China	A cross-sectional study	This study aimed to investigate the relationship between geriatric diseases and microelements.	Total (n) = 69 (n=18) obese individuals (n=51) non-obese individuals
	Xu, G.Y. [36]	China	A cross-sectional study	This study aimed to explore the relationship between trace elements and hyperlipidemia.	Total (n) = 52 (n=19) obese individuals (n=33) non-obese individuals
Zinc	Yu, S. M. [37]	China	A cross-sectional study	This study aimed to explore the relationship between trace elements copper and zinc and hyperlipidemia in coronary heart disease.	Total (n) = 95 (n=54) obese individuals (n=41) non-obese individuals
	Li, J. R. [38]	China	A cross-sectional study	This study aimed to clarify the correlation between blood glucose, abnormal blood lipid metabolism, and serum zinc, copper, and magnesium levels in patients with type 2 DM.	Total (n) = 65 (n=23) obese individuals (n=42) non-obese individuals
	Pei, W. J. [39]	China	A cross-sectional study	This study aimed to investigate the correlation between serum zinc, copper, magnesium, prostacyclin, and thromboxane in patients with hyperlipidemia.	Total (n) = 77 (n=42) obese individuals (n=35) non-obese individuals
	He, B. P. [40]	China	A cross-sectional study	This study aimed to investigate the relationship between serum zinc (Zn) and copper (Cu) contents and apolipoprotein a-i (Apo A-i) and Apo B (Apo B).	Total (n) = 74 (n=44) obese individuals (n=30) non-obese individuals
	Zhuang, Y.Y [41]	China	A cross-sectional study	This study aimed to explore the micronutrients and the occurrence and development of cardiovascular and cerebrovascular diseases.	Total (n) = 70 (n=30) obese individuals (n=40) non-obese individuals
	Yan, X. M. [42]	China	A cross-sectional study	This study aimed to explore the changes of Cu and Zn contents in HTC, HTG, HTCHTG, and control group and the correlation between Cu, Zn, Cu/Zn contents and TG and TC contents.	Total (n) = 320 (n=160) obese individuals (n=160) non-obese individuals
	Yang, C. J. [43]	China	A cross-sectional study	This study aimed to explore the relationship between trace elements zinc, copper, iron, chromium, selenium, and different types of dyslipidemia.	Total (n) = 187 (n=87) obese individuals (n=100) non-obese individuals
	Yao, Y. F. [44]	China	A cross-sectional study	This study aimed to investigate the relationship between serum zinc and copper levels and blood lipid in patients with type 2 diabetes mellitus complicated with coronary heart disease.	Total (n) = 95 (n=60) obese individuals (n=35) non-obese individuals
Iron	Karen G. N. [45]	USA	A prospective cohort study	This study aimed to investigate the association between weight status, as measured by BMI, and iron deficiency in a nationally representative sample of children and adolescents.	Total (n) = 9698 13.7% were at risk for overweight 10.2% were overweight
	Bowen Z. [46]	China	A cross-sectional study	The study aims to establish the relationships between iron status with apolipoproteins and lipid ratios.	Total (n) = 7540
	Tussing-Humphreys, L. M. [47]	USA	A cross-sectional study	The study aims to assess the relationship between iron status and excess adiposity, inflammation, menarche, diet, and so on.	female adolescents between ages 12 and 17 years (n=210)
Iron	Nuria A. [48]	Spain	A prospective cohort study	This study aimed to assess the relationship between elevated iron levels and lipid peroxidation in Caucasian adults residing in the northeastern Mediterranean region of Spain.	Total (n) = 300 (n=150) cases (n=150) controls
	Tang, L. H. [49]	China	A cross-sectional study	This study aims to explore the relationship between SF and MS by measuring sF levels in MS patients.	Total (n) = 300 (n=150) cases (n=150) controls
	Zhuang, Y. Y. [41]	China	A cross-sectional study	This study aimed to explore the micronutrients and the occurrence and development of cardiovascular and cerebrovascular diseases.	Total (n) = 70 (n=30) obese individuals (n=40) non-obese individuals
	Fan, L. M. [50]	China	A cross-sectional study	To explore the relationship between serum ferritin and body mass index (BMI), waist circumference/hip ratio (WHR), blood pressure, blood lipids, fasting insulin (FINS), and fasting blood glucose (FPS).	Total (n) = 100 (n=50) obese individuals (n=50) non-obese individuals
	Yang, C. J. [43]	China	A cross-sectional study	This study aimed to explore the relationship between trace elements zinc, copper, iron, chromium, selenium, and different types of dyslipidemia.	Total (n) = 187 (n=87) obese individuals (n=100) non-obese individuals
	Zhang, L. C. [51]	China	A cross-sectional study	To explore the role of plasma ferritin (SF) in the pathogenesis of hyperlipidemia and to study the levels of SF and oxidative stress in patients with hyperlipidemia to provide a specific basis for their treatment and prognosis.	Total (n) = 200 (n=100) obese individuals (n=100) non-obese individuals
	Lee, H. S. [52]	Korea	A cross-sectional study	This study was undertaken to examine the relationship between the serum ferritin level and depression in Korean male adults concerning the classification of overall obesity.	Total (n) = 55 (n=28) obese individuals (n=27) non-obese individuals
	Yerlikaya, F. H. [34]	America	A cross-sectional study	The study aimed to investigate the relationship between plasma microRNA expression levels, which are associated with lipid metabolism, and serum trace element levels in patients with primary hyperlipidemia.	Total (n) = 83 (n=46) obese individuals (n=37) non-obese individuals
Selenium	Joachim B. [53]	USA	A prospective cohort study	The study aimed to investigate the effects of selenium intake on the lipid profile in selenium-replete populations.	Total (n) = 5452
	Zhao, Z. [54]	USA	A prospective cohort study	The objective was to reveal the impacts and mechanisms of a moderately high Se and a high fat intake on lipid metabolism in Gpx1 knockout (KO) and wild-type (WT) mice.	The KO and WT mice (males, 12-wk-old)
	Wen J. [55]	China	A cross-sectional study	This study aimed to investigate the associations of serum selenium concentrations with lipid concentrations and dyslipidemia.	Total (n) = 8198 rural Chinese
	Chen C. [56]	China	A longitudinal study	The study aimed to examine the associations of selenium status with changes in lipid levels in a 7-year follow-up of an elderly Chinese cohort.	Total (n) = 140 elderly Chinese
Selenium	Frederik C. [57]	Denmark	A cross-sectional study	This study aimed to investigate the association between Se and cholesterol concentrations.	Total (n) = 491 (n 124), 200 (n 122) or 300 (n 119) μg Se-enriched yeast placebo-yeast tablets (n 126)
	Hassan B. [58]	Iran	A cross-sectional study	This study aimed to evaluate the effect of selenium supplementation during pregnancy on cord blood selenium content and lipid profile.	Total (n) = 166 eligible women who were randomized to receive 100 mg of selenium
	Zhuang, Y. Y. [41]	China		This study aimed to explore the micronutrients and the occurrence and development of cardiovascular and cerebrovascular diseases.	Total (n) = 70 (n=30) obese individuals (n=40) non-obese individuals
	Yang, C. J. [43]	China	A cross-sectional study	This study aimed to explore the relationship between trace elements zinc, copper, iron, chromium, selenium, and different types of dyslipidemia.	Total (n) = 187 (n=87) obese individuals (n=100) non-obese individuals
	Moon, S. [59]	Korea	A cross-sectional study	This study aimed to ascertain the relationship between selenium and DM.	Total (n) = 3406 (n=633) obese individuals (n=2773) non-obese individuals
	Yerlikaya, F. H. [34]	America	A cross-sectional study	The study aimed to investigate the relationship between plasma microRNA expression levels, which are associated with lipid metabolism, and serum trace element levels in patients with primary hyperlipidemia.	Total (n) = 83 (n=46) obese individuals (n=37) non-obese individuals
Copper	Jixuan M. [60]	China	A cohort study	This study focused on investigating associations between urinary copper and blood lipid profiles, and exploring the potential role of systemic inflammation in such relationships.	Total (n) = 4812 participants aged 18-80
	Junxi C. [61]	China	A prospective cohort study	This study aimed to investigate the relationship between copper exposure and blood lipid metabolism in the women population.	Total (n) =35 women in northern China
	Cheng, W. L. [35]	China	A cross-sectional study	This study aimed to investigate the relationship between geriatric diseases and microelements.	Total (n) = 69 (n=18) obese individuals (n=51) non-obese individuals
	Yu, S. M. [37]	China	A cross-sectional study	This study aimed to explore the relationship between trace elements copper and zinc and hyperlipidemia in coronary heart disease.	Total (n) = 95 (n=54) obese individuals (n=41) non-obese individuals
	Pei, W. J. [39]	China	A cross-sectional study	This study aimed to investigate the correlation between serum zinc, copper, magnesium, prostacyclin, and thromboxane in patients with hyperlipidemia.	Total (n) = 77 (n=42) obese individuals (n=35) non-obese individuals
Copper	He, B. P. [40]	China	A cross-sectional study	This study aimed to investigate the relationship between serum zinc (Zn) and copper (Cu) contents and apolipoprotein a-i (Apo A-i) and Apo B (Apo B).	Total (n) = 74 (n=44) obese individuals (n=30) non-obese individuals
	Li, X. H. [30]	China	A cross-sectional study	To further clarify the correlation between blood glucose and dyslipidemia and the levels of serum zinc, copper, and magnesium in type 2 diabetes mellitus patients.	33 diabetes mellitus patients and 33 non-diabetes mellitus healthy controls
	Yan, X. M. [42]	China	A cross-sectional study	This study aimed to explore the changes of Cu and Zn contents in HTC, HTG, HTCHTG, and control group and the correlation between Cu, Zn, Cu/Zn contents and TG and TC contents.	Total (n) = 320 (n=160) obese individuals (n=160) non-obese individuals
	Yang, C. J. [43]	China	A cross-sectional study	This study aimed to explore the relationship between trace elements zinc, copper, iron, chromium, selenium, and different types of dyslipidemia.	Total (n) = 187 (n=87) obese individuals (n=100) non-obese individuals
	Yao, Y. F. [44]	China	A cross-sectional study	This study aimed to investigate the relationship between serum zinc and copper levels and blood lipid in patients with type 2 diabetes mellitus complicated with coronary heart disease.	Total (n) = 95 (n=60) obese individuals (n=35) non-obese individuals
	Yerlikaya, F. H. [34]	America	A cross-sectional study	The study aimed to investigate the relationship between plasma microRNA expression levels, which are associated with lipid metabolism, and serum trace element levels in patients with primary hyperlipidemia.	Total (n) = 83 (n=46) obese individuals (n=37) non-obese individuals
	Ngu, Y. J. [29]	China	A cross-sectional study	To clarify associations of exposure to essential and non-essential metals with body composition and risks of obesity and MetS.	150 Taiwanese adults (100 men and 50 women)
Calcium	Jaak J. [62]	Estonia	A prospective cohort study	The aim was to investigate the possible association of dietary calcium intake with adiposity, insulin resistance, and adipocytokine values in adolescent boys.	Total (n)= 123 adolescent boys aged 13-15 years
	Farjam G. [63]	Iran	A prospective cohort study	The present investigation aimed to study the influx and efflux of Ca^2+^ into and out of the cells during adipogenesis.	Total (n)=4 young women aged between 25 and 35 years
	Leila S. [18]	Iran	A prospective cohort study	This study aimed to evaluate the association between diastolic blood pressure (DBP) and 25(OH.)D concentrations with lipid profile in overweight and obese women.	Total (n)= 236 overweight and obese women
Chrome	Yang, C. J. [43]	China	A cross-sectional study	This study aimed to explore the relationship between trace elements zinc, copper, iron, chromium, selenium, and different types of dyslipidemia.	Total (n) = 187 (n=87) obese individuals (n=100) non-obese individuals
Chrome	Yerlikaya, F. H. [34]	America	A cross-sectional study	The study aimed to investigate the relationship between plasma microRNA expression levels, which are associated with lipid metabolism, and serum trace element levels in patients with primary hyperlipidemia.	Total (n) = 83 (n=46) obese individuals (n=37) non-obese individuals
	Ngu, Y. J. [29]	China	A cross-sectional study	To clarify associations of exposure to essential and non-essential metals with body composition and risks of obesity and MetS.	150 Taiwanese adults (100 men and 50 women)
Manganese	Yerlikaya, F. H [34]	America	A cross-sectional study	The study aimed to investigate the relationship between plasma microRNA expression levels, which are associated with lipid metabolism, and serum trace element levels in patients with primary hyperlipidemia.	Total (n) = 83 (n=46) obese individuals (n=37) non-obese individuals
	Ngu, Y. J. [29]	China	A cross-sectional study	To clarify associations of exposure to essential and non-essential metals with body composition and risks of obesity and MetS.	150 Taiwanese adults (100 men and 50 women)
Cadmium	Zhou, Z. [64]	China	A cross-sectional study	This study aimed to test the hypothesis that exposure to cadmium is related to the prevalence of dyslipidemia.	Total (n) = 1489 (n=987) obese individuals (n=502) non-obese individuals
	Asgary, S. [65]	Iran	A cross-sectional study	This study aimed to evaluate serum concentrations of lead (s-Pb), mercury (s-Hg), and cadmium (s-Cd) in patients with CAD in comparison with those of healthy individuals.	Total (n) = 127 (n=63) obese individuals (n=64) non-obese individuals
	Yerlikaya, F. H. [34]	America	A cross-sectional study	The study aimed to investigate the relationship between plasma microRNA expression levels, which are associated with lipid metabolism, and serum trace element levels in patients with primary hyperlipidemia.	Total (n) = 83 (n=46) obese individuals (n=37) non-obese individuals
	Ngu, Y. J. [29]	China	A cross-sectional study	To clarify associations of exposure to essential and non-essential metals with body composition and risks of obesity and MetS.	150 Taiwanese adults (100 men and 50 women)

## Data Availability

The data of the study are available from the corresponding author, upon reasonable request.

## LIST OF ABBREVIATIONS

CNKI: China National Knowledge Infrastructure
SMD: Standardized Weighted Mean Difference

## STANDARDS OF REPORTING

PRISMA guidelines and methodology were followed.

## SUPPLEMENTARY MATERIAL

PRISMA checklist is available as supplementary material on the publisher’s website along with the published article.
